# Bovine piroplasmosis‐anaplasmosis and clinical signs of tropical theileriosis in the plains of Djurdjura (north Algeria)

**DOI:** 10.1002/vms3.305

**Published:** 2020-06-17

**Authors:** Hocine Ziam, Tahar Kernif, Khelaf Saidani, Rabah Kelanemer, Zoheir Hammaz, Dirk Geysen

**Affiliations:** ^1^ Institue of veterinary Sciences Saad Dahlab University Blida 1 Ouled Yaich 9015 Blida Algeria; ^2^ Laboratory of Biotechnology, Environment and Health Saad Dahlab University Blida 9015 Algeria; ^3^ Institute of Tropical Medicine Department of Biomedical Sciences Nationalestraat 155 Antwerp 2000 Belgium; ^4^ Laboratory of Parasitic Eco‐epidemiology and Population Genetics Pasteur Institute of Algeria Algiers Dely‐Brahim Algeria; ^5^ High School of Food Sciences and Food Industry Oued Smar Algiers 16270 Algeria

**Keywords:** Algeria, *Anaplasma marginale*, *Babesia bigemina*, *Babesia bovis*, clinical signs, Djurdjura, *Theileria annulata*

## Abstract

The study was conducted during tick activity season over a period of 5 years in the Djurdjura Plains, Algeria. A total of 299 cattle (Holstein, Montbeliard, Fleckvieh and crossbred animals) with clinical signs were included in this study. A total of 171 animals were found positive for at least one pathogen by Giemsa‐stained blood smears examination *Theileria annulata* (136/299, 45.5%), *Babesia bovis* (14/299, 4.7%), *B. bigemina* (3/299, 1.0%) and *Anaplasma marginale* (12/299, 4.0%) were identified. Six animals were co‐infected by *T. annulata* and *A. marginale*. Although no ticks were collected from diseased animals, clinical signs in cattle were hyperthermia (120/136, 88.3%), gluttony followed by anorexia (113/136, 83.1%), lymph node enlargement (99/136, 72.8%), anaemia (82/136, 60.3%), icterus (58/136, 42.6%) and haemoglobinuria (36/136, 26.5%). Gluttony followed by anorexia was considered highly suggestive of an incubation of tropical theileriosis as shown by a higher receptivity index (IR = 0.89–1). This clinical sign is evident in young Montbeliard and young Holstein males with anaemia (IR = 1) and icterus (IR = 0.78–0.81) which is earlier than haemoglobinuria (IR = 0.51–0.54). The incidence of *T. annulata* was maximum in July (*n* = 57), as well as *B. bovis* (*n* = 6) and *A. marginale* (*n* = 13). These results highlight the preponderance of tropical theileriosis in north‐central Algeria, where gluttony followed by anorexia is probably a prodromal symptom during the incubation period of the disease.

## Graphical abstract

The cells transformed by *Theileria* act like cancer and consume a lot of glucose. Cattle incubated with *Theileria annulata* show gluttony followed by anorexia, prodromal clinical signs for early diagnosis of tropical theileriosis.

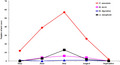

## 1 INTRODUCTION

Tick‐borne diseases are of great economic importance in livestock in many regions of the world. Ticks transmit many pathogens, including bacteria, viruses, rickettsia and protozoa resulting in important infections of both humans and animals (Wikel, 2018). Tick‐borne diseases like the protozoa *Theileria* and *Babesia* spp. induce piroplasmosis and the bacterium *Anaplasma* (genogroup of *Ehrlichia*) results in anaplasmosis (Camus & Uilenberg, 2010).

In Algeria, tropical theileriosis is the most prevalent infection in cattle, followed by anaplasmosis and babesiosis, with incidences of 57.2%, 6.2% and 3.8%, respectively (Ziam et al., 2016; Ziam & Benaouf, 2004). Of these three enzootic diseases, tropical theileriosis remains by far the dominant summer disease in Algeria.

Symptoms of theileriosis is expressed mainly by gluttony followed by anorexia, febrile generalized lymphadenitis and anaemia (Narladkar, Digraskar, & Potekar, 2005; Ziam et al., 2016). Babesiosis is characterized by a haemolytic anaemia, icterus, haemoglobinuria and shock. Anaplasmosis is caused by an intraerythrocytic rickettsial pathogen, inducing hyperthermia, progressive anaemia, weight loss and drop in milk yield (Song et al., 2018). These diseases induce major economic losses due to weight loss, decreased meat and milk production, the prohibitive cost of treatment, abortions, high morbidity and mortality (Figueroa, L'hostis, & Camus, 2010; Gharbi et al., 2011).

In epidemiological studies of piroplasmosis and anaplasmosis, serology and PCR are excellent diagnostic tools with good sensitivity and specificity. However, PCR could not discriminate carrier from diseased animals and serology might not be positive during the disease course particularly in early clinical cases. That is why these tests could not be used for confirmation of disease cases (Ait‐Hamou et al., 2012; Bilgic et al., 2016; El Haj et al., 2002; Ziam et al., 2015). Nevertheless, suspicion of the disease is based on the association of epidemiological elements (period of specific tick activity, the type of livestock, state of the stables in the case of tropical theileriosis presence of cracks and crevices) and clinical signs associated, confirmed by positive Giemsa stained blood or lymph node smears (Ziam, Saidani, & Aissi, 2017). Unfortunately, the remoteness and/or the lack of well‐equipped veterinary laboratories complicate correct diagnosis of piroplasmosis and anaplasmosis.

As differential diagnosis between these three tick‐borne diseases might be difficult, especially in relapsing tropical theileriosis cases or in some reinfection cases with a new genotype which are associated with a transient lymph node enlargement. Identification of prodromal symptoms of the disease may help clinicians to better identify the disease outcome and initiate early treatment before the onset of hyperthermia and generalized swelling of lymph nodes, severe anaemia, icterus and petechia, decreasing the probability of the animal recovery. In Algeria, tropical theileriosis is the dominant cattle summer disease (Ayadi, Gharbi, & Benchikh‐Elfegoun, 2017; Rouina, 1984; Ziam et al., 2017). The objective of this work was to highlight the clinical incidence of bovine anaplasmosis and piroplasmosis, especially tropical theileriosis, and associated early clinical signs in cattle of exotic and local breeds in the plains of Djurdjura, Algeria.

## 2 MATERIALS AND METHODS

### 2.1 Study area

**FIGURE 1:**
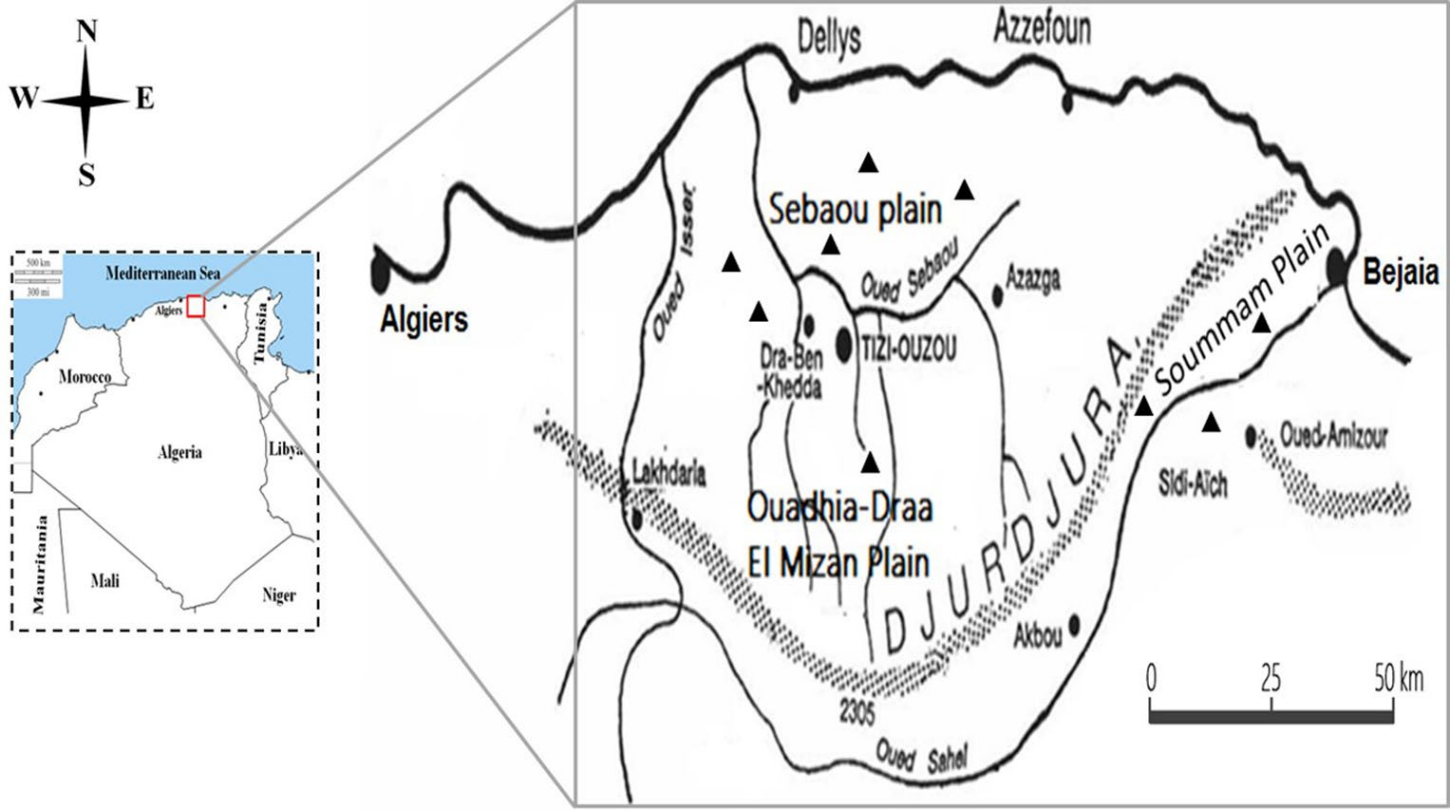
Geographic localization of the region of Djurdjura, North Algeria, triangle shows the localities where bovines were sampled

The study was conducted in the plains of Djurdjura located in the North Central Algeria. It is a vast area, of 5,144,2 km^2^, between longitudes 7°08' to 8°37' E and latitude 36°43' to 37°7' N, this region has a landscape interspersed with valleys and mountainous regions (Figure 1).

### 2.2 Climate and vegetation

Djurdjura region has a Mediterranean climate. Its annual rainfall varies between 600 in the south and 1.200 mm in north with a relative humidity of 65%–75% during the summer season. The region is sub‐humid to humid with hot and dry summers and cool and rainy winters. Temperature reaches 35°C in summer and drops to 5°C in winter, the northwest winds generate heavy rainfall accompanied by cold waves (Amroun, Bensidhoum, Delattre, & Gaubert, 2013; Meddour, Meddour‐Sahar, Derridj, & Gehu, 2010). According to Meddour et al. (2010), cedar forests of the Atlas and cork oak represents 42% of the total area of the region, and more than 3,800 plant species of plants were recorded. Natural grasslands constitute the bulk of the diet of cattle (Meddour et al., 2010).

### 2.3 Animals

This study was carried out between 2004 and 2008, during tick activity period, from May to September (Gharbi et al., 2020). Moreover, anaplasmosis is also transmitted mechanically by haematophagous vectors (*Tabanus*, *Stomoxys*…). Suspected cattle with piroplasmosis or anaplamosis, presented to the veterinary clinics (*n* = 7), were sampled in the Djurdjura area from 270 farms having a herd size of more than 15 animals. Cattle were reared under a semi‐intensive system and consisted of exotic breed Holstein, Montbeliard, Fleckvieh and crossbred. Grass, hay, straw and crop residues are the main diet of the animals, supplemented with concentrates.

### 2.4 Diagnosis and treatment

A total of 299 cattle showing clinical signs of either piroplasmosis or anaplasmosis, such as hyperthermia, lymph node enlargement, agalactia, haemoglobinuria, icterus and gluttony followed by pronounced anorexia were included in the study. Clinical examination, Giemsa‐stained blood smears, pathogen identification and treatment of animals were done as described previously (Ziam et al., 2017). Briefly, blood smears were made from the ear vein of each suspected sick animal, fixed in methanol, stained with Giemsa and examined under a microscope using an oil‐immersion ×100 objective. At least, 100 microscopic fields were carefully examined for blood forms of *Anaplasma* spp., *Theileria* spp. and *Babesia* spp. An animal was considered positive when showing the presence of one or more of these pathogens. *Theileria annulata* positive animals were treated with buparvaquone (ButalexTM, Schering‐Plough) at a dose of 2.5 mg/kg; whereas imidocarb dipropionate (Imizol®, Merck) at a dose of 6.6 mg/kg was used to treat babesiosis and anaplasmosis. After treatment, there was no further follow‐up for pathogen presence.

### 2.5 Index of receptivity (IR)

A correlation between an observed symptom and the number of animals truly affected by *T. annulata*, *B. bovis*, *B. bigemina* and *A. marginale* was evaluated using the index of receptivity (IR) as proposed by Ziam et al., (2016). The IR (varies from 0 to 1) was calculated by relating the total frequency of a clinical sign to the number of animals actually affected by *T. annulata, B. bovis, B. bigemina* or *A. marginale* showing this sign. The IR is more important when it rises or reaches 1 and less important when it falls to zero.

### 2.6 Statistical analysis

Mixed‐effect logistic regression was used to evaluate the influence of age, sex and breed on the incidence of various diseases and to compare their incidences. The comparisons of the frequencies of clinical signs were performed using the chi‐square test. Prioritization of clinical signs was performed according to the method of classification tree and regression (Dahms, 2004).

## 3 RESULTS

### 3.1 Clinical signs

Table 1 highlights the specific frequencies of different clinical signs observed on 171 diseased cattle according to each pathogen. A total of 119 (69.6%) diseased animals expressed gluttony for up to 24 hr followed by anorexia. The latter clinical sign might be complicated by acidosis, when animals were fed with concentrate or an overload indigestion when fed with hay and/or straw. *Theileria annulata* positive animals (*n* = 136) showed hyperthermia (120/136, 88.3%), gluttony followed by anorexia (113/136, 83.1%), adenitis with enlarged pre‐scapular and pre‐crural lymph nodes (99/136, 72.8%) varying in size from thumb thick to a mandarin, anaemia (82/136, 60.3%), icterus (58/136, 2.6%) and haemoglobinuria (36/136, 26.5%). The numbers of animals positive for *B. bovis*, *B. bigemina* and *A. marginale* were low and therefore the frequency of these specific clinical signs was high (Table 1). In dairy cows, a total of 97 lactating cows showed sudden agalactia. The presence of dead ticks was always confirmed through inspection of the tick‐fixation sites like ear conch, baleen, ano‐genital region, legs and the inguinal region.

**TABLE 1:** Frequency of clinical signs in 171 cattle suspected of piroplasmosis and anaplasmosis (N, %±*SE*) from the plains of Djurdjura, North Algeria

	*Theileria annulata*	*Babesia bovis*	*Babesia bigemina*	*Anaplasma marginale*	*Theileria annulata/Anaplasma marginale*
Hyperthermia	120 (88.3 ± 2.76)	13 (92.8 ± 6.9)	3 (100)	10 (83.3 ± 10.76)	5 (83.3 ± 15.22)
Lymph node enlargement	99 (72.8 ± 3.83)	8 (57.2 ± 13.20)	2 (66.7 ± 27.23)	8 (66.7 ± 13.61)	4 (66.7 ± 19.25)
Anaemia	82 (60.3 ± 4.19)	7 (50.0 ± 13.36)	1 (33.3 ± 27.20)	9 (75.0 ± 12.50)	5 (83.3 ± 15.22)
Gluttony/anorexia	113 (83.1 ± 3.22)	0.0	0.0	0.0	6 (100)
Icterus	58 (42.6 ± 4.24)	3 (21.41 ± 0.96)	2 (66.7 ± 27.23)	3 (25.0 ± 12.5)	4 (66.7 ± 19.25)
Haemoglobinuria	36 (26.5 ± 3.77)	0.0	0.0	1 (8.3 ± 7.96)	3 (50.0 ± 20.41)

Abbreviations: %, Percentage of cattle expressed each symptom; Gluttony/anorexia: gluttony followed by anorexia; *N*, Number of cattle expressed each symptom.

### 3.2 Microscopic examination

The different pathogens observed in blood were *Theileria annulata*, *Babesia bovis*, *B. bigemina* and *Anaplasma marginale* (Table 2). These pathogens were identified in 171 (57.2%) of total 299 bovines enrolled in the study. Most of these pathogens have been found in Holsteins (49/76, 64.5%), Fleckvieh (15/26, 57.7%), crossbred (30/53, 56.6%) and Montbeliard (77/144, 53.5%) (*p* < .05). However, in the recruited cattles, Holsteins were significantly more infected by *T. annulata*, *B. bovis* and *A. marginale* (*p* < .01) compared to the other three breeds (Table 2). The frequency of pathogens was comparable between animals aged of less than 1 year (10/21, 47.6%) versus older than 1 year (161/278, 57.9%) (*p* < .001) but was higher in females (157/252, 62.3%) compared to males (14/47, 29.8%) (*p* < .001).

**TABLE 2:** Frequency of infections in cattle suspected of piroplasmosis and anaplasmosis (N, %±*SE*) from the plains of Djurdjura, North Algeria

Epidemiological parameters	Suspected cattle	*Theileria annulata*	*Babesia bovis*	*Babesia bigemina*	*Anaplasma marginale*	*Theileria annulata*/*Anaplasma marginale*	Total
Breed							
Holstein	76	35 (46.0 ± 5.71)	7 (9.23.20)	0	5 (6.6 ± 2.80)	2 (2.6 ± 1.70)	49 (64.5 ± 5.10)
Montbeliard	144	63 (43.7 ± 4.10)	3 (2.1 ± 1.17)	1 (0.7 ± 0.66)	7 (4.8 ± 1.73)	3 (2.1 ± 1.17)	77 (53.5 ± 4.90)
Fleckvieh	26	14 (53.8 ± 9.74)	0.0	1 (3.8 ± 3.77)	0.0	0.0	15 (57.7 ± 9.64)
Crossbred	53	24 (45.3 ± 6.83)	4 (7.5 ± 3.57)	1 (1.9 ± 1.83)	0.0	1 (1.9 ± 1.83)	30 (56.6 ± 6.78)
Ages							
≤1 an	21	9 (42.8 ± 10.70)	0.0	0.0	1 (4.7 ± 4.59)	0.0	10 (47.6 ± 10.80)
>1 an	278	127 (45.7 ± 2.94)	14 (5.0 ± 1.27)	3 (1.1 ± 0.61)	11 (3.9 ± 1.12)	6 (2.2 ± 0.86)	161(57.9 ± 2.95)
Sexes							
Males	47	12 (25.5 ± 5.96)	1 (2.1 ± 2.04)	1 (2.1 ± 2.04)	0.0	0.0	14 (29.8 ± 6.63)
Females	252	124 (49.2 ± 3.11)	13 (5.2 ± 1.37)	2 (0.8 ± 0.51)	12 (4.7 ± 1.32)	6 (2.4 ± 0.91)	157 (62.3 ± 3.01)
Total	299	136 (45.5 ± 2.58)	14 (4.7 ± 1.17)	3 (1.0 ± 0.56)	12 (4.0 ± 1.12)	6 (2.0 ± 0.76)	171(57.2 ± 2.85)

Abbreviations: %, Percentage of positives cattle; *N*, Number of positives cattle.

Among 171 positive blood smears, *T. annulata* single infection was identified in 136 cattle and associated infections with *A. marginale* in six cases. Single *A. marginale* was found in 12 cases (4.0%), *Babesia bovis* (14 cases) and the three cases of *B. bigemina* were identified as single infection (Table 2). Among the positive Holsteins cattle (*n* = 49), 71.4% (*n* = 35) were infected by *T. annulata*, 14.3% (*n* = 7) by *B. bovis,* and 10.2% (*n* = 5) by *A. marginale*. Two (4.1%) animals were simultaneously infected by *T. annulata* and *A. marginale*, whereas the cumulative incidence of *A. marginale* was 14.3% (*n* = 7). Of the 77 Montbeliard cattle with positive blood smears, *T. annulata* was found in 81.8% (*n* = 63), *B. bovis* in 3.9% (*n* = 3), *B. bigemina* in 1.3% (*n* = 1) and *A. marginale* in 9.1% (*n* = 7) of the animals. Co‐infections by *T. annulata* and *A. marginale* were found in 3.9% (*n* = 3) of the animals and the cumulative infection rate of *A. marginale* was 13.0% (*n* = 10). In positive Fleckvieh cattle (*n* = 15), 14 animals were infected by *T. annulata*, and only one by *B. bigemina* no co‐infections were observed. Among the positive crossbred cattle (*n* = 30), *T. annulata* was identified in 80.0% (*n* = 24), *B. bovis* in 13.4% (*n* = 4), *B. bigemina* in 3.3% (*n* = 1) and one animal showed a co‐infection by *T. annulata* and *A. marginale*.

There was no statistically difference between infection rates by *B. bigemina* in the three breeds Montbeliard, Fleckvieh and crossbred (*p* > .05). The incidence of infection by each pathogen was higher in adults than in young animals as well as in females (*p* < .001) compared to males (Table 2). Among 128 negative animals, 93 expressed clinical signs of tropical theileriosis but were negative for the presence of blood pathogens. Hyperthermia and lymph node enlargement were the most dominant symptoms and three out of 93 bovines showed gluttony in the absence of parasites.

### 3.3 Chronology of different pathogens

**FIGURE 2:**
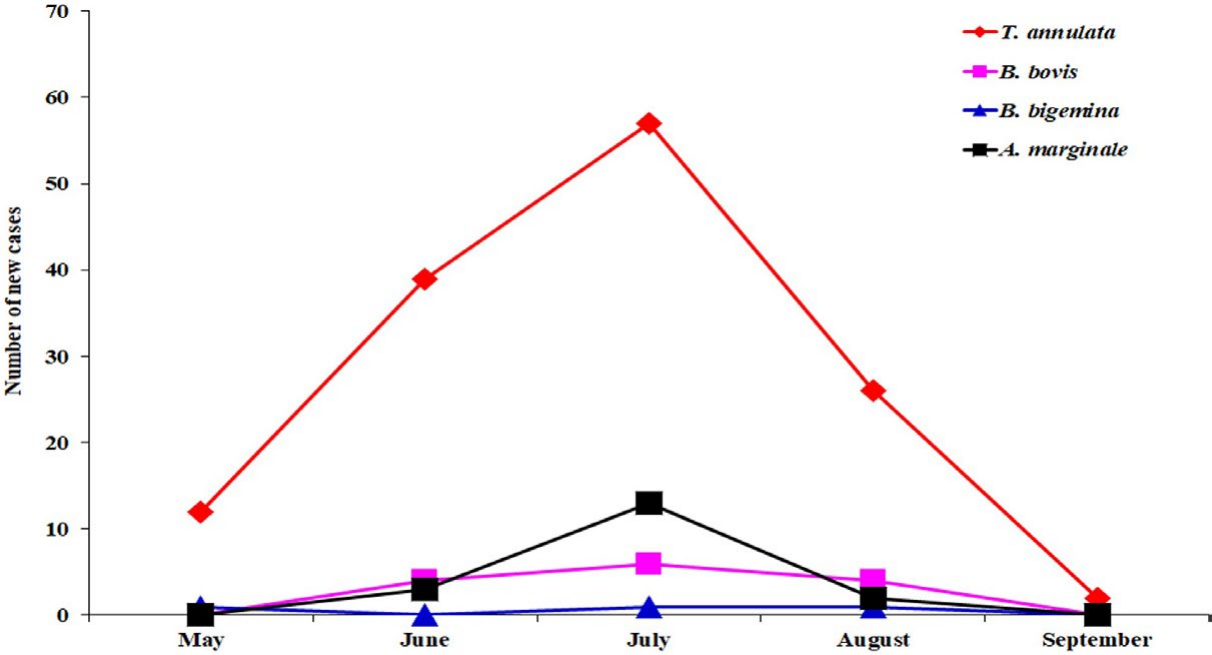
Incidence of piroplasmosis and anaplasmosis from the plains of Djurdjura, North Algeria

The chronological distribution of clinical cases for each pathogen during the study period (May to September) is shown in Figure 2. The overall incidences for each pathogen were significantly different (*p* < .001). Tropical theileriosis increased linearly from May to June with an incidence of 12 to 39 new clinical cases, respectively, and reaching a peak of 57 cases in July. Thereafter, the disease decreased towards 26 cases in August and dropped to two cases in September. Statistical analysis indicated that the rate of tropical theileriosis in July was significantly higher (*p* < .001) than those of May, June, August and September and the incidence during June was also significant (*p* < .001), compared to those of May, August and September and between August and September (*p* < .001). The number of clinical cases due to *A. marginale* and *B. bovis* (*p* < .05–.001) was higher in July than during the other months of the summer season (Figure 2). Only three animals were infected by *B. bigemina* during May, July and August.

### 3.4 Index of receptivity

The IR of the various clinical signs, for each identified pathogen, in piroplasmosis and anaplasmosis suspected cattle are presented in Table 3. The four breeds cattle with piroplasmosis and anaplasmosis exhibit gluttony followed by anorexia (IR = 0.71), icterus (IR = 0.67) and haemoglobinuria (IR = 0.40). It was found that Holstein cattle with piroplasmosis and anaplasmosis had a higher IR for anaemia (IR = 0.89).

**TABLE 3:** Receptivity index based on clinical signs of cattle with piroplasmosis and anaplasmosis form the plains of Djurdjura, North Algeria

Symptoms	*Theileria annulata*	*Babesia bovis*	*Babesia bigemina*	*Anaplasma marginale*	*Theileria annulata*/ *Anaplasma marginale*	Total
Hyperthermia						
Holstein (*n* = 71)	2.02 (71/35)	10.14 (71/7)	−(71/0)	14.2 (71/5)	35.5 (71/2)	1.44 (71/49)
Montbeliard (*n* = 126)	2 (126/63)	42 (126/3)	126 (126/1)	18 (126/7)	42 (126/3)	1.63 (126/77)
Fleckvieh (*n* = 26)	1.85 (26/14)	−(26/0)	26 (26/1)	−(26/0)	−(26/0)	1.73 (26/15)
Crossbred (*n* = 46)	1.91(46/24)	11.50 (46/4)	46 (46/1)	−(46/0)	46 (46/1)	1.53 (46/29)
Total (*n* = 269)	1.97 (269/136)	19.21 (269/14)	86.66 (269/3)	22.41 (269/12)	44.83 (269/6)	1.57 (269/171)
Adenitis						
Holstein (*n* = 57)	1.62 (57/35)	8.14 (57/7)	−(57/0)	11.4 (57/5)	28.5 (57/2)	1.16 (57/49)
Montbeliard (*n* = 97)	1.53 (97/63)	32.33 (97/3)	97 (97/1)	13.85 (97/7)	32.33 (97/3)	1.25 (97/77)
Fleckvieh (*n* = 23)	1.64 (23/14)	−(23/0)	23 (23/1)	−(23/0)	−(23/0)	1.53 (23/15)
Crossbred (*n* = 39)	1.62 (39/24)	9.75 (39/4)	39 (39/1)	−(39/0)	39 (39/1)	1.30 (39/30)
Total (*n* = 216)	1.58 (216/136)	15.42 (216/14)	72 (216/3)	18 (216/12)	36 (216/6)	1.26 (216/171)
Anaemia						
Holstein (*n* = 44)	1.25 (44/35)	6.28 (44/7)	−(44/0)	8.80 (44/5)	22.00 (44/2)	0.89 (44/49)
Montbeliard (*n* = 87)	1.38 (87/63)	29.00 (87/3)	87 (87/1)	12.42 (87/7)	29.00 (87/3)	1.12 (87/77)
Fleckvieh (*n* = 21)	1.5 (21/14)	−(21/0)	21 (21/1)	−(21/0)	−(21/0)	1.40 (21/15)
Crossbred (*n* = 37)	1.54 (37/24)	9.25 (37/4)	37 (37/1)	−(37/0)	37.00 (37/1)	1.23 (37/30)
Total (*n* = 189)	1.38 (189/136)	13.5 (189/14)	63 (189/3)	15.75 (189/12)	31.5 (189/6)	1.10 (189/171)
Gluttony/anorexia						
Holstein (*n* = 27)	0.77 (27/35)	3.85 (27/7)	−27/0)	5.4 (27/5)	13.5 (27/2)	0.55 (27/49)
Montbeliard (*n* = 59)	0.93 (59/63)	19.66 (59/3)	59.00 (59/1)	8.42 (59/7)	19.66 (59/3)	0.76 (59/77)
Fleckvieh (*n* = 14)	1.00 (14/14)	−(14/0)	14.00 (14/1)	−(14/0)	−(14/0)	0.93 (14/15)
Crossbred (*n* = 22)	0.91 (22/24)	5.50 (22/4)	22.00 (22/1)	−(22/0)	22.00 (22/1)	0.73 (22/30)
Total (*n* = 122)	0.89 (122/136)	8.71 (122/14)	40.66 (122/3)	10.16 (122/12)	20.33 (122/6)	0.71 (122/171)
Icterus						
Holstein (*n* = 23)	0.65 (23/35)	3.28 (23/7)	−(23/0)	4.6 (23/5)	11.50 (23/2)	0.46 (23/49)
Montbeliard (*n* = 56)	0.88 (56/63)	18.66 (56/3)	56.00 (56/1)	8.00 (56/7)	18.66 (56/3)	0.72 (56/77)
Fleckvieh (*n* = 15)	1.07 (15/14)	−(15/0)	15.00 (15/1)	−(15/0)	−(15/0)	1.00 (15/15)
Crossbred (*n* = 21)	0.87 (21/24)	5.25 (21/4)	21.00 (21/1)	−(21/0)	21.00 (21/1)	0.70 (21/30)
Total (*n* = 115)	0.84 (115/136)	8.21(115/14)	38.33 (115/3)	9.58 (115/12)	19.16 (115/6)	0.67 (115/171)
Haemoglobinuria						
Holstein (*n* = 15)	0.42 (15/35)	2.14 (15/7)	−(15/0)	3.00 (15/5)	7.50 (15/2)	0.30 (15/49)
Montbeliard (*n* = 32)	0.50 (32/63)	10.66 (32/3)	32.00 (32/1)	4.57 (32/7)	10.66 (32/3)	0.41 (32/77)
Fleckvieh (*n* = 7)	0.50 (7/14)	−(7/0)	7.00 (7/1)	−(7/0)	−(7/0)	0.46 (7/15)
Crossbred (*n* = 16)	0.66 (16/24)	4.00 (16/4)	16.00 (16/1)	−(16/0)	16.00 (16/1)	0.53 (16/30)
Total (*n* = 70)	0.51 (70/136)	5.00 (70/14)	23.33 (70/3)	5.83 (70/12)	11.66 (70/6)	0.40 (70/171)

Abbreviations: Gluttony/anorexia, gluttony followed by anorexia; IR, Index of receptivity corresponding to the ratio of overall frequency of clinical sign/ number of animals positive for blood smear.

The symptoms recorded in the four cattle breeds when infected by *T. annulata* consisted in gluttony followed by anorexia (IR = 0.89), icterus (IR = 0.84) and haemoglobinuria (IR = 0.51) (Table 3). Gluttony was confirmed in 96.7% of cattle infected with *T. annulata* as a specific clinical sign in all breeds when the classification tree and regression was used (IR = 0.89) (Table 3). The IR of icterus was also high in the investigated breeds (IR = 0.84) (Table 3) infected by *T. annulata*. This symptom associated with gluttony followed by anorexia is mostly seen in tropical theileriosis. The IR of haemoglobinuria was 0.51 (Table 3) for all breeds infected by *T. annulata*. These symptoms associated with previous clinical signs gave a good indication that the animal might be suffering of tropical theileriosis. Because of the low incidence of infections with *B. bovis*, *B. bigemina* and *A. marginale* the IRs are significantly greater than unit (Table 3).

Gluttony followed by anorexia is a specific and common symptom in young Holstein, Montbeliard and Fleckvieh as well as in Holstein, Montbeliard, Fleckvieh and crossbred cows infected by *T. annulata* (Table 4). There is no difference in such symptoms between males and females of Montbeliard and crossbred animals (IR = 0.90–1) (Table 4). *T. annulata* infections give rise to anaemia which appears to be specific in young Holstein and Montbeliard as well as in Holstein males (IR = 1). Icterus was a common symptom for young Holstein and Fleckvieh as well as for adult Holstein, Montbeliard, crossbred and Fleckvieh females (IR = 0.60–1) whereas haemoglobinuria was a specific clinical sign in Montbeliard, Fleckvieh and crossbred cows infected by *T. annulata* (IR = 0.50–0.58) (Table 4).

**TABLE 4:** Receptivity index according to age and sex of cattle with tropical theileriosis from the plains of Djurdjura, North Algeria

Epidemiological parameters	Hyperthermia	Adenitis	Anaemia	Gluttony/anorexia	Icterus	Haemoglobinuria
Age category						
Young (≤1 an) (*n* = 9)	2.33 (21/9)	1.11 (10/9)	1.22 (11/9)	0.77 (7/9)	1.44 (13/9)	0.22 (2/9)
Holstein (*n* = 1)	8.00 (8/1)	3.00 (3/1)	1.00 (1/1)	1.00 (1/1)	1.00 (1/1)	−(0/1)
Montbeliard (*n* = 7)	1.57 (11/7)	0.71 (5/7)	1.00 (7/7)	0.71 (5/7)	1.42 (10/7)	0.14 (1/7)
Fleckvieh (*n* = 1)	2.00 (2/1)	2.00 (2/1)	2.00 (2/1)	1.00 (1/1)	1.00 (1/1)	−(0/1)
Crossbred (*n* = 0)	−(3/0)	−(2/0)	−(2/0)	−(0/0)	−(1/0)	−(1/0)
Adults (>1 an) (*n* = 127)	1.98 (252/127)	1.62(206/127)	1.98 (252/127)	0.88 (112/127)	0.81 (103/127)	0.51 (63/127)
Holstein (*n* = 34)	2.02 (69/34)	1.61 (55/34)	1.26 (43/34)	0.76 (26/34)	0.64 (22/34)	0.44 (15/34)
Montbeliard (*n* = 56)	2.05 (115/56)	1.64 (92/56)	1.42 (80/56)	0.91 (51/56	0.82 (46/56)	0.51 (29/56)
Fleckvieh (*n* = 13)	1.92 (25/13)	1.69 (22/13)	1.53 (20/13)	1.00 (13/13)	1.15 (15/13)	0.53 (7/13)
Crossbred (*n* = 24)	1.79 (43/24)	1.54 (37/24)	1.45(35/24)	0.91 (22/24)	0.83(20/24)	0.62 (15/24)
Sexes						
Males (*n* = 12)	3.33 (40/12)	2.75 (33/12)	1.91 (23/12)	1.16(14/12)	1.66 (20/12)	0.25 (3/12)
Holstein (*n* = 3)	3.33 (10/3)	3.00 (9/3)	1.00 (3/3)	1.66 (5/3)	0.33 (1/3)	−(0/3)
Montbeliard (*n* = 7)	2.85 (20/7)	2.42 (17/7)	1.85 (13/7)	1.00 (7/7)	1.85 (13/7)	0.28 (2/7)
Fleckvieh (*n* = 0)	−(3/0)	−(3/0)	−(3/0)	0.00 (0/0)	−(3/0)	0.00 (0/0)
Crossbred (*n* = 2)	3.50 (7/2)	2.00 (4/2)	2.00 (4/2)	1.00 (2/2)	1.50 (3/2)	0.50 (1/2)
Females (*n* = 124)	1.84 (229/124)	1.47 (183/124)	1.33 (166/124)	0.84 (105/124)	0.78 (95/124)	0.54 (68/124)
Holstein (*n* = 32)	2.42 (51/21)	1.50 (48/32)	1.28 (41/32)	0.68 (22/32)	0.68 (22/32)	0.46(15/32)
Montbeliard (*n* = 56)	1.89 (106/56)	1.421 (80/56)	1.32 (74/56)	0.91 (51/56)	0.60 (43/56)	0.53 (30/56)
Fleckvieh (*n* = 14)	1.64 (23/14)	1.42 (20/14)	1.28 (18/14)	1.00 (14/14)	1.00 (14/14)	0.50 (7/14)
Crossbred (*n* = 22)	1.77 (39/22)	1.59 (35/22)	1.50(33/22)	0.90 (20/22)	0.81 (12/22)	0.68 (7/22)

Abbreviations: Gluttony/anorexia, gluttony followed by anorexia; IR, Index of receptivity corresponding to the ratio of overall frequency of clinical sign/ number of animals positive for blood smear.

## 4 DISCUSSION

*Theileria annulata* is an intracellular parasite diverting cellular biochemical signalling pathways to ensure its development in the host cell. They infect B‐cells and other monocytes transforming them into lymphoblast and monoblast cells (Dobbelaere & Rottenberg, 2003). *Theileria*‐transformed cells act like cancer cells and are known to consume a lot of glucose (Haidar, Echebli, Ding, Kamau, & Langsley, 2015; Haidar, Whitworth, et al., 2015). It has been shown that cows with a starting *T. annulata* infection increase their food intake, particularly high‐energy foods (Ziam et al., 2016). This study indicated that 122 animals out of 299 suspected cases showed gluttony for up to 24 hr, and a large proportion of these animals were naturally infected with *T. annulata* (Table 4). This gluttony seems to be the expression of an infectious state in which the parasite interferes with its host cell metabolism to its advantage. Thus, these animals need external supply of energy to satisfy the high glucose uptake by *Theileria*‐transformed cells (Haidar, Echebli, et al., 2015; Haidar, Whitworth, et al., 2015). These results confirm our preliminary observations and indicate that gluttony in cattle might be a pathognomonic symptom for early diagnosis of tropical theileriosis. Similar observation was reported in India (Narladkar et al., 2005).

Our data pointed out that the gluttony followed by anorexia was associated with other symptoms related to the evolution of the disease. Among them, hyperthermia, lymph node enlargement, anaemia, icterus and haemoglobinuria, similar to previously reported results (Darghouth, Kilani, & Bouattour, 2003; Rouina, 1984; Ziam et al., 2017).

The gluttony shown by cattle infected with *T. annulata* highlights the prodromal marker nature of this clinical sign especially when followed by anorexia. This was evident in *T. annulata* infected young Holstein and Fleckvieh as well as adult Montbeliard and Fleckvieh (IR = 0.84–1). This clinical expression of tropical theileriosis is also found in Holsteins, Montbeliard and crossbred cattle as well as in female Montbeliard and Fleckvieh (IR = 0.91–1) similar to the results reported previously (Ziam et al., 2016).

Gharbi, Latrach, Sassi, and Darghouth (2012) stated that haemolytic anaemia syndrome is a specific clinical sign of tropical theileriosis, and confirms our results that anaemia (IR = 1, see Table 4) is a specific symptom in young Holstein and Montbeliard cattle with tropical theileriosis (Gharbi et al., 2012; Ziam et al., 2016).

Icterus (IR = 0.84) and haemoglobinuria (IR = 0.51) have been well observed and are suggestive symptoms of tropical theileriosis. Icterus was seen in the early stages of tropical theileriosis as confirmed by an IR = 0.84 and haemoglobinuria appears towards the final phase of the disease (IR = 0.51) similar to the results reported by Darghouth et al. (2003). This symptom is the result of haemolysis, induced by merozoïtes of *T. annulata*, followed by excretion of haemoglobin in urines (Gharbi et al., 2012).

The other most frequently reported symptoms were hyperthermia (40.5°C) and lymph node enlargement, which are relatively non‐specific, because relapse of tropical theileriosis or reinfection with a new genotype of the parasite induces a transient superficial lymph node enlargement, difficult to assess during palpation, making clinical diagnosis of the disease difficult.

For practitioners as well as for farmers, tropical theileriosis is often fatal disease for old dairy cattle showing atypical clinical symptoms. In situations of cows with post‐natal stress or animals re‐infected with a new parasite strains, an atypical disease course might result with fleeting lymph node enlargement and subclinical parasitaemia, taken misleadingly as babesiosis (Ziam et al., 2017). Therefore, inclusion of lymph node smears is a necessary diagnostic tool, but breeders are often reluctant to lymph node puncture.

This study revealed that 27 cattle showed clinical signs of tropical theileriosis, but were found positive for *B. bovis*, *B. bigemina* and *A. marginale*. Medical treatment of these animals remains difficult as drug treatment (requiring usually a theilericide and babesicide by lack of proper diagnosis) is excessively expensive and farmers prefer to slaughter such animals.

In this study, hyperthermia and anaemia were the common clinical signs of babesiosis and anaplasmosis. According to Camus and Uilenberg (2010), there is neither haemoglobinuria nor icterus in anaplasmosis, apart from intermittent hyperthermia. However, 12 Flickviehs and 16 crossbreds showed symptoms of piroplasmosis and/or anaplasmosis, whereas blood smear examination of such animals was negative. Because of the low sensitivity of the Giemsa stained blood smear during babesiosis associated with low parasitaemia, and the importance of differentiation between *A. centrale* and *A. marginale*, this observation emphasizes the importance of repeated microscopic examination, especially in negative animals (Camus & Uilenberg, 2010).

According to the International Office of Epizootic (2018), the symptoms of tropical theileriosis are similar to those of East Coast fever, but animals may show anaemia and icterus with yellowish coloration of the mucous membranes of the eyes and gums. Chronic constipation reflects an inadequacy in bile salts production as a consequence of haemolytic anaemia and weak digestion. In this investigation, all animals with gluttonous symptoms were positive for tropical theileriosis and these presented also haemoglobinuria in 23.4%.

In this survey, young animals were not infected with *Babesia* spp. According to Figueroa et al. (2010), this might be due to a natural resistance in calves aged 6 to 9 months, the absence of exposure to exophilic ticks associated with low infestation rates in calves and finally due to colostral immunity (Figueroa et al., 2010).

The high rates of tropical theileriosis in this study are similar to those previously observed in the wilayates of Annaba and El Tarf in eastern Algeria (Ziam et al., 2016). The high prevalence of clinical cases due to tropical theileriosis in adult exotic breeds suggests endemic instability or relapsing disease due to stress. By studying this phenomenon in dairy cattle over four summers, (Darghouth et al., 2003) argued that stress due to milk production, pregnancy and calving are essentially implicated in the susceptibility to infection and subsequently in the expression of clinical symptoms.

Even though no live ticks were found on the animals during our study period, lesions testifying earlier infestations by ticks were found in the genito‐anal, inguinal region and auricular conch. Only dry ticks, probably dead following acaricidal treatments, were found on animals. Our attempts to collect these ticks were unsuccessful. *Hyalomma scupense* is present throughout Northern Algeria and implicated in the transmission of *T. annulata* (Gharbi & Darghouth, 2014; Ziam et al., 2017).

The diagnosis of bovine piroplasmosis and anaplasmosis requires a good knowledge of its seasonal occurrence, clinical signs and epidemiology. This work reveals four haemopathogens in the plain of Djurdjura, namely *T. annulata*, *A. marginale*, *B. bovis* and *B. bigemina*. With an infection rate of 79.5%, tropical theileriosis remains the most dominant tick‐borne disease in the region. Clinical signs start with gluttony over 24 hr followed by a severe anorexia preceding the more clinical signs like hyperthermia, generalized lymph node enlargement, anaemia and icterus. In the absence of medication, anorexia persists with a pathophysiological progression of the disease until the animal dies. Further large‐scale research should be carried out to determine the prevalence and epizootiology of blood pathogens, in particular *T. annulata*, *B. bovis*, *B. bigemina*, *B. divergens*, *A. marginale*, *A. centrale*, *Ehrlichia* spp. by morphological identification, serology and molecular technique.

## conflict of interest

The authors declare no conflict of interest.

## AUTHOR CONTRIBUTION

**Hocine Ziam:** Conceptualization; Data curation; Investigation; Methodology; Supervision; Writing‐original draft; Writing‐review & editing. **Kernif Tahar:** Data curation; Formal analysis; Methodology; Validation; Writing‐original draft; Writing‐review & editing. **SAIDANI Khelaf:** Formal analysis; Investigation; Methodology; Software; Validation; Writing‐original draft; Writing‐review & editing. **KELANEMER Rabah:** Data curation; Investigation; Methodology; Writing‐original draft; Writing‐review & editing. **HAMMAZ Zoheir:** Data curation; Investigation; Methodology; Writing‐original draft; Writing‐review & editing. **Dirk Geysen:** Conceptualization; Funding acquisition; Methodology; Supervision; Validation; Writing‐original draft; Writing‐review & editing.
